# Effects of Selected Essential Oils on *Listeria monocytogenes* in Biofilms and in a Model Food System

**DOI:** 10.3390/foods12101930

**Published:** 2023-05-09

**Authors:** Suzana Vidaković Knežević, Slobodan Knežević, Jelena Vranešević, Sneẑana Ž. Kravić, Brankica Lakićević, Sunčica Kocić-Tanackov, Nedjeljko Karabasil

**Affiliations:** 1 Scientific Veterinary Institute “Novi Sad”, 21000 Novi Sad, Serbia; slobodan.knezevic@niv.ns.ac.rs (S.K.); jelenababic@niv.ns.ac.rs (J.V.); 2 Faculty of Technology Novi Sad, University of Novi Sad, 21000 Novi Sad, Serbia; sne@uns.ac.rs (S.Ž.K.); suncicat@uns.ac.rs (S.K.-T.); 3 Institute of Meat Hygiene and Technology, 11040 Belgrade, Serbia; brankica.lakicevic@inmes.rs; 4 Faculty of Veterinary Medicine, University of Belgrade, 11000 Belgrade, Serbia; nedja@vet.bg.ac.rs

**Keywords:** antimicrobial activity, antibiofilm activity, minced pork meat, *Listeria monocytogenes*, oregano essential oil, thyme essential oil

## Abstract

The composition of 18 essential oils was determined using gas chromatography–mass spectrometry, and their antilisterial activity was evaluated by the disk diffusion method, followed by the determination of the minimum inhibitory and minimum bactericidal concentrations. The most active essential oils were oregano, thyme, cinnamon, winter savory, and clove, with MIC values ranging from 0.09 to 1.78 µL/mL. We investigated the biofilm-forming potential of *Listeria monocytogenes* on polystyrene at 5 °C, 15 °C, and 37 °C in three different media. The formation of biofilm was found to be dependent on the temperature and the availability of nutrients. After treatment with selected essential oils, the reduction in biofilm biomass was in the range of 32.61% and 78.62%. Micromorphological changes in the *L. monocytogenes* treated by oregano and thyme essential oils were observed in the form of impaired cell integrity and cell lyses by using scanning electron microscope. Oregano and thyme essential oils (MIC and 2MIC) significantly (*p* < 0.05) reduced the population of *L. monocytogenes* in minced pork meat during storage at 4 °C. In conclusion, the obtained results indicated the good activity of some selected essential oils on *L. monocytogenes*, with bacteriostatic, bactericidal, and antibiofilm effects at very low concentrations.

## 1. Introduction

The genus *Listeria* includes 22 species [1], of which *L. monocytogenes* and *L. innocua* are the most prevalent [2]. *L. monocytogenes* is a Gram-positive, facultative anaerobe, rod-shaped bacteria, about 0.5 µm in width and 1–1.5 µm in length [3], with the ability to multiply in a wide temperature range (2–45 °C), a wide pH range (4.6–9.5), and water activity values of at least 0.92 [4,5,6].

*L. monocytogenes* is widely distributed in nature. Although ready-to-eat food is considered the main route of transmission of *L. monocytogenes* [7], this bacterium is often present in production facilities on equipment and surfaces that come into contact with food in the form of biofilm, as well as in raw foods of plant and animal origin [8]. The development of biofilm prevents *L. monocytogenes* from adverse environmental factors, such as the traditional chemical antimicrobial agents, thermal treatment, UV exposure, and pH shifts, among others [9].

Epidemiological data show that 98% of listeriosis outbreaks are associated with three *L. monocytogenes* serotypes: 1/2a, 1/2b, and 4b [4,10], despite the fact that 13 serotypes are capable of causing infections in humans. Listeriosis in humans is primarily the result of consuming contaminated food. The minimum infectious dose for humans has not been determined, but research shows that it ranges between 10^2^ and 10^9^ CFU, and depends on the immune status of the host. The incubation period lasts from 11 to 70 days (an average of 21 days) [10]. In the early stages of listeriosis, non-specific symptoms appear, such as flu symptoms (fatigue, cold, headache, weakness). This bacterium can cause mild gastroenteritis or severe invasive diseases, such as septicemia, meningitis, encephalitis, and abortion and death [3,6]. Those most susceptible to listeriosis are immunocompromised people, the elderly, pregnant women, and infants [4,7].

The increase in bacterial resistance to antibiotics, the lack of new drug development, and increased concern about the use of synthetic additives continues to be a pressing challenge that drives the search for promising alternatives against *L. monocytogenes* [11]. Therefore, the use of natural preservatives, such as essential oils (EOs), has increased. Essential oils are complex mixtures of compounds that are synthesized by different plant parts (roots, stems, leaves, flowers, fruits, and seeds). The antibacterial properties of EOs are mainly assigned to individual components (e.g., carvacrol, thymol, *p*-cymene, and cinnamaldehyde) [12].

The effect of EOs on bacteria is reflected in the inhibition of their growth or the destruction of the bacterial cell [13,14,15] through different specific mechanisms of action. The interaction of EOs with the bacteria cells causes the degradation of the cell wall, damage to the cytoplasmic membrane, and damage to membrane proteins. This damage induces the leakage of the cell contents, the coagulation of the cytoplasm, and the depletion of the proton motive force, all causing cell death [16].

However, the antibacterial effect of EOs in biofilm and food model systems is affected by many factors which can lead to reduced efficiency in comparison with in vitro studies.

The aim of this research was to determine the antimicrobial and antibiofilm activity of different EOs and their influence on the growth of *L. monocytogenes* isolated from different stages of meat production and meat products both in vitro and in a model food system.

## 2. Materials and Methods

### 2.1. Bacterial Strains

The *L. monocytogenes* strains (L1 isolated from fermented sausage; L5, L17, L18 and UB2-01-2 (1) isolated from surfaces in the meat industry) used in this research were isolated from the meat industry. These strains were stored as frozen culture at −80 °C in a tryptic soy broth (TSB) (Biokar Diagnostics, Beauvais, France) medium containing 20% glycerol until examination. The strains were cultivated on nutrient agar (Biokar Diagnostics, Beauvais, France) slants for 24 h at 37 °C before the experiments.

### 2.2. Essential Oils

For this experiment, eighteen EOs were selected (Supplementary Materials Table S1). Commercially available EOs of basil (*Ocimum basilicum*), black pepper (*Piper nigrum*), cassumunar ginger (*Zingiber cassumunar*), cinnamon (*Cinnamomum zeylanicum Nees*), clove (*Syzygium aromaticum* L.), curry plant (*Helichrysum italicum*), fennel (*Foeniculum vulgare*), lavender (*Lavandula angustifolia*), lemon (*Citrus limonum*), myrtle (*Myrtus communis*), oregano (*Origanum vulgare*), rosemary (*Rosmarinus officinalis*), sage (*Salvia officinalis*), thyme (*Thymus vulgaris*), (TerraCo d.o.o., Novi Sad, Republic of Serbia), garden angelica (*Angelica archangelica*), hyssop (*Hyssopus officinalis*), winter savory (*Satureja montana*), and yarrow (*Achillea millefolium*) (Siempreviva oils, Niš, Republic of Serbia) were chemically analyzed with a gas chromatograph (GC 7890B, Agilent Technologies) coupled to a mass spectrometer (MS 5977A, Agilent Technologies) as previously described by Vidaković Knežević et al. [17]. Briefly, a HP-5MS capillary column (30 m × 0.25 mm i.d., film thickness 0.25 µm; Agilent Technologies) was used. The GC oven temperature was initially set to 70 °C for 2 min, and then gradually increased to 220 °C at the rate of 4 °C/min and held at 220 °C for 10 min. The mass spectrometer was operated with an ionization energy of 70 eV. The carrier gas was helium (1 mL/min), the injector temperature was 250 °C, the injection volume was 1 µL (10% hexane solution), and the split ratio was 1:80.

### 2.3. Disk Diffusion Method

To evaluate the antilisterial effects of EOs, the disk diffusion method was performed [18]. Briefly, plates of Mueller-Hinton agar (Biokar Diagnostics, Beauvais, France) were surfaced-layered with a bacteria suspension (1.0 × 10^8^ CFU/mL) by sterile swabs. Amounts of 5 and 10 µL of each EO were placed on a sterile filter paper disc of 6 mm diameter (HiMedia Laboratories Pvt. Ltd., Mumbai, India) and incubated at 37 °C for 24 h. After incubation, the diameters of growth-inhibiting zones around the discs, including the diameter of the discs, were measured in mm. Discs with no EOs were used as the negative control. The experiments were performed in triplicate for each EO. EOs that presented no inhibition zones were excluded from the following experiments.

### 2.4. Broth Microdilution Method

The minimum inhibitory concentration (MIC) and minimum bactericidal concentration (MBC) values of EOs were determined by the broth microdilution method according to the guideline of [19], slightly modified, as per [20]. Briefly, an aliquot of 100 µL Muller-Hinton broth (Oxoid, Basingstoke, UK) was inoculated into a 96-well sterile U-bottomed polystyrene microtiter plate (Nuova Aptaca SRL, Canelli, Italy). Next, 100 µL of the tested EO was added to the first well, and twofold serial dilutions were performed in the test wells. Upon completion, 10 μL of the *L. monocytogenes* suspension (10^8^ CFU/mL) was added to each well and mixed thoroughly. The EOs were diluted in 10% dimethylsulfoxide (DMSO) (Lach-Ner sro, Czech Republic). Sterile Mueller-Hinton broth and EOs were used as the negative control, while the Mueller-Hinton broth inoculated with *L. monocytogenes* was used as the positive control. After 24 h incubation at 37 °C, the content of each well was spread with a sterile wire loop onto Mueller-Hinton agar and incubated for another 24 h at 37 °C. The lowest concentrations without visible growth onto Mueller-Hinton agar were defined as MBCs, while the lowest concentrations that showed slightly visible growth on the Mueller-Hinton agar were defined as MICs. The experiments were performed in duplicates for each EO.

### 2.5. Biofilm Formation

The ability of *L. monocytogenes* strains to form biofilm was tested in a 96-well sterile flat-bottomed polystyrene microtiter plate (Sarstedt, Nümbrecht, Germany). The strains were sub-cultured in tryptic soy broth (TSB) (Oxoid, United Kingdom) at 37 °C for 24 h, and then diluted to a 1:40 ratio in TSB, Luria-Bertani broth (LB) (Oxoid, United Kingdom) and meat broth (MB) (Oxoid, UK). Each well was inoculated with 200 µL of bacterial suspension and incubated at 5 °C, 15 °C, and 37 °C for 48 h. After incubation, the wells were discarded and washed with physiological saline three times (250 μL/well) to remove non-adherent bacterial cells. The plates were then air dried, and the adhered bacterial cells were fixed by adding 250 μL/well of 96% ethanol (Reahem, Srbobran, Republic of Serbia) for 20 min. Next, the ethanol was removed and 250 μL of 0.3% crystal violet (Fluka, Sigma-Aldrich, Germany) dye was added for 20 min. The microtiter plates were rinsed under tapped water and air dried. Finally, 250 μL/well of ethanol was added to solubilize the dye, and the OD_550_ was recorded using a microplate reader (SYS Expert Plus Microtitration Reader, Biochrom, Cambridge, Great Britain). Tests were performed in triplicate for each isolate, with four repetitions. The cut-off OD_C_ was defined as three standard deviations above the mean OD of the negative control. *L. monocytogenes* strains were classified according to Stepanović et al. [21] as strong biofilm producer (OD > (4 × OD_C_)), moderate biofilm producer ((2 × OD_C_) < OD ≤ (4 × OD_C_)), weak biofilm producer (OD_C_ ≤ OD ≤ (2 × OD_C_)), and no biofilm producer (OD ≤ OD_C_).

### 2.6. Biofilm Reduction

The biofilm formation of *L. monocytogenes* strains was performed as described in the section above (Section 2.5). After removing the non-adherent bacterial cells with physiological saline (3 × 250 μL/well), the biofilms were treated with the MBC concentrations of EOs. Namely, 200 µL of EOs solution in TSB were added to the wells and incubated at 37 °C and 15 °C for 48 h. As previously described (Section 2.5), the plates were washed and stained with crystal violet solution. ODs were measured at 550 nm. Tests were performed in triplicate for each isolate with four repetitions, and the inhibition percentages were calculated by Formula (1) [22]:

[(OD_GROWTH CONTROL_ − OD_SAMPLE_)/OD_GROWTH CONTROL_] × 100, (1)

### 2.7. Scanning Electron Microscopy (SEM)

Scanning electron microscopy was used to observe the effects of the EOs on the bacterial cells. The bacterial adhesion was conducted in 12-well plates, each containing a sterile SS 304 stainless steel coupon (1 × 1 × 0.1 cm). An aliquot (100 µL) of selected bacterial suspension (UB2-01-2 (1)) prepared in Section 2.5 was added to the stainless steel coupons and incubated at 37 °C for 3 h. After incubation, the coupons were washed with physiological saline (3 mL). The control well contained 2 mL LB broth, while the wells with treated bacterial cells contained MIC of oregano and thyme EOs (0.18 µL/mL and 0.36 µL/mL, respectively). After 24 h incubation at 37 °C, the coupons were washed three times with physiological saline, and fixed in 4% glutaraldehyde overnight at 5 °C, followed by washing with physiological saline and dehydration in graded ethanol series (30%, 50%, 60%, 70%, 90%) for 5 min at each concentration. After this process, the coupons were submerged in 96% ethanol three times for 10 min each. Finally, the coupons were air dried and gold coated in a Baltic Scan Sputter Coater SCD 005 (WD = 50 mm, 90 s, 30 mA) and examined with a scanning electron microscope (JMS SEM 6460 LV, operating at an accelerating voltage of 25 kV).

### 2.8. Minced Pork Meat Preparation

*Quadriceps femoris* pork muscle was aseptically rendered with a No. 4 grinding disc. Samples of minced pork meat were weighted (10 g) and stored in sterile Petri dishes. Minced pork meat was examined for any contamination by *L. monocytogenes* prior to inoculation with *L. monocytogenes* UB2-01-2 (1) and the addition of oregano and thyme EOs. Minced pork meat samples were inoculated with the *L. monocytogenes* strain to obtain a final concentration of ca. 10^4^–10^5^ CFU/g. To ensure the proper distribution of *L. monocytogenes,* the inoculated minced pork meat samples were homogenized with a sterile glass rod. The oregano and thyme EOs were then added at concentrations of 0.18, 0.36, and 0.72 µL/g, which were obtained as MIC and 2MIC in the broth microdilution method. Once again, the minced pork meat samples were homogenized. All samples were stored at 4 ± 1 °C and analyzed on days 0, 1, 2, 3, and 4.

### 2.9. Microbiological Analysis

Each sample was homogenized for 2 min in 90 mL sterilized peptone water (Biokar Diagnostics, Beauvais, France), decimal diluted, cultured on Agar Listeria according to Ottaviani and Agosti (ALOA, CM1084, Oxoid) agar plates, and incubated at 37 °C for 48 h according to the ISO method [23]. The experiment was performed in triplicate, and each experiment consisted of two repetitions.

### 2.10. Statistical Analysis

The data were analyzed using statistical software R version 3.2.2 (R Foundation for Statistical Computing, Vienna, Austria). An Analysis of variance (ANOVA), followed by Duncan’s test, was performed to determine the statistical difference between the samples. Differences were considered significant at *p* < 0.05.

## 3. Results and Discussion

### 3.1. Chemical Composition of Essential Oils

The chemical compositions of the 18 EOs (basil, black pepper, cassumunar ginger, cinnamon, clove, curry plant, fennel, garden angelica, hyssop, lavender, lemon, myrtle, oregano, rosemary, sage, thyme, winter savory, and yarrow) are provided in Tables S2–S4 (Supplementary Materials). The main components of basil EO were estragole (69.52%) and linalool (24.77%). Those of black pepper EO were β-pinene (19.31%), limonene (13.93), and sabinene (13.55%). Sabinene (38.17%) was, along with terpinene-4-ol (35.90%), the main component of cassumunar ginger EO. Cinnamon and clove EOs were rich in cinnamaldehyde (74.93%) and eugenol (85.14%), respectively. The three main compositions of curry plant EO were caryophyllene (21.48%), neryl acetate (18.15%), and β-himachalene (13.34%). Fennel and garden angelica EOs were rich in anethole (88.42%), and β-phellandrene (41.57%), respectively. Cis-pinocamphone (27.42%) was the main component of hyssop EO, while linalyl acetate (25.33%), and linalool (23.88%) were the main components of lavender EO. The major components of lemon, myrtle, and oregano EOs were limonene (79.72%), α-pinene (35.47%), and carvacrol (81.00%), respectively. The two main components of rosemary EO were α-pinene (28.23%), and borneol (24.87%). The sage EO was rich in linalyl acetate (56.41%). The major components of thyme EO were *p*-cymene (40.91%), and thymol (40.36%), while the main components of winter savory and yarrow EOs were carvacrol (50.45%), and sabinene (22.70%), respectively. Similar findings have been reported in previous studies [24,25,26,27,28,29].

### 3.2. Antilisterial Activity of Essential Oils

The disk diffusion method was performed as a screening method (Table 1), and EOs with antilisterial activity were further selected for MICs and MBCs. The EOof fennel showed no inhibition of *L. monocytogenes* strains, and therefore was excluded from further testing. Essential oils of basil, rosemary, curry plant, sage, garden angelica, yarrow, and hyssop showed no inhibition of some *L. monocytogenes* strains regardless of the applied amount of EOs. *L. monocytogenes* strains showed a wide range of sensitivity to black pepper (7.00–16.33 mm), cassumunar ginger (7.00–21.00 mm), clove (11.33–17.33 mm), lavender (8.00–15.00 mm), lemon (7.67–16.33 mm), and myrtle (8.00–15.67 mm) EOs. The highest inhibition zones were obtained for EOs of cinnamon, oregano, thyme and winter savory. The antilisterial activity of thyme EO could be due to the high *p*-cymene and thymol content. The *p*-cymene is a precursor of carvacrol, and causes swelling of the cytoplasmic membrane [30], while thymol causes its permeability [31] and the loss of intracellular components, including ATP [32]. As with thymol, carvacrol (the major component in oregano and winter savory EOs) acts via the same mechanism on bacterial cells [33]. The activity of cinnamon EO is based on the presence of cinnamaldehyde, an aromatic aldehyde that inhibits the synthesis of essential enzymes in bacterial cells, and causes damage to the bacterial cell wall [34,35].

### 3.3. MICs and MBCs Determination

**Figure 1:**
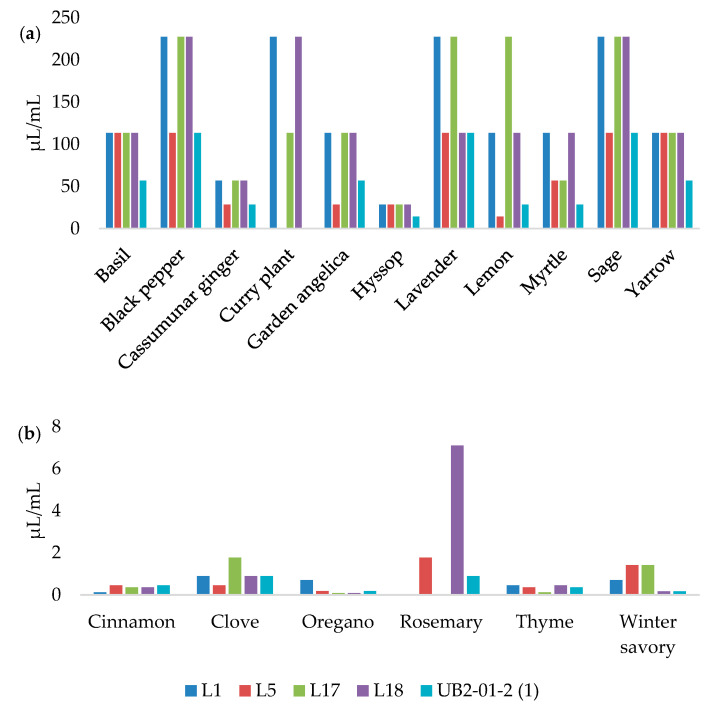
Minimum inhibitory concentrations of basil, black pepper, cassumunar ginger, curry plant, garden angelica, hyssop, lavender, lemon, myrtle, sage, and yarrow; (**a**) cinnamon, clove, oregano, rosemary, thyme, and winter savory; (**b**) essential oils acting against the *L. monocytogenes* strains.

Figure 1 presents the MIC values of EOs against *L. monocytogenes* strains.

**Figure 2:**
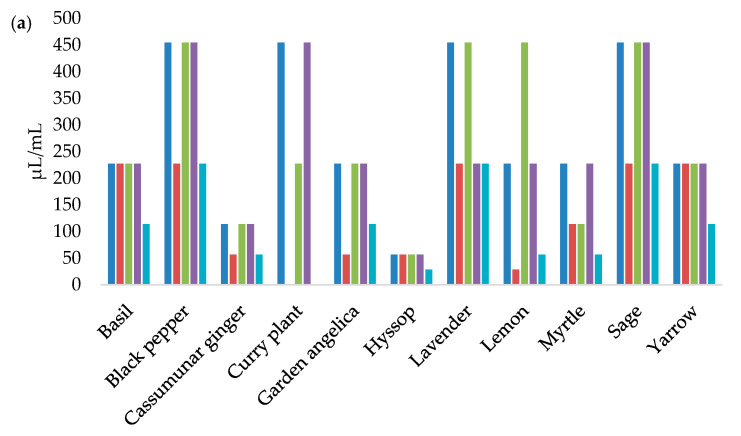

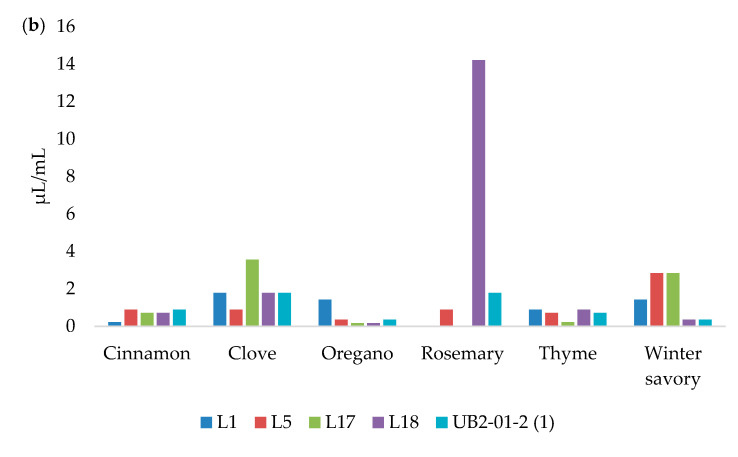
Minimum bactericidal concentrations of basil, black pepper, cassumunar ginger, curry plant, garden angelica, hyssop, lavender, lemon, myrtle, sage, and yarrow; (**a**) cinnamon, clove, oregano, rosemary, thyme, and winter savory; (**b**) essential oils acting against the *L. monocytogenes* strains.

According to Figure 2, the MBC values of the EOs are twice the MIC values.

The EOs with the largest inhibition zones were mostly those with the lowest MIC values. The broth microdilution method confirmed that the oregano, thyme, cinnamon, and winter savory EOs had remarkable antilisterial effects, inhibiting all of the *L. monocytogenes* strains in very small concentrations. The MIC values of oregano EO ranged from 0.09 to 0.72 µL/mL, while the MIC values of thyme and cinnamon EOs ranged from 0.12 to 0.45 µL/mL. The MIC values for winter savory and clove EOs were very similar, at 0.17–1.42 µL/mL and 0.45–1.78 µL/mL, respectively. Similar results were reported by Burt [33], and Mazzarrino et al. [36]. However, it was found that the MIC values do not always correlate with the inhibition zones due to differences in the growth of microorganisms in liquid and solid media, the degree of exposure of microorganisms to EOs, the solubility of EOs, and their origin [37]. This is best demonstrated by rosemary EO, which did not inhibit the growth of two *L. monocytogenes* isolates by the disk diffusion method. However, the MICs of this EO started from 0.89 µL/mL, classifying this EO as an exceptional antilisterial agent. The ability of certain EOs to have a better effect on bacterial cells in a liquid medium has been noted before [38,39]. The broth microdilution method is generally more sensitive, and allows for a quantitative determination of the antibacterial activity of EOs [37].

### 3.4. Biofilm Formation

The biofilm formation by *L. monocytogenes* strains used in this study was evaluated under three different temperatures (5 °C, 15 °C, and 37 °C) and three different growth media (TSB, MB, and LB broth). The obtained results are shown in Table 2.

The cut-off values of 0.108, 0.133, and 0.123 were established to consider biofilm formation at 37 °C in TSB, MB, and LB broth, respectively. At 15 °C, the established cut-off values were 0.173, 0.167, and 0.157 in the TSB, MB, and LB broth, respectively. The biofilm formation at 5 °C was considered according to cut-off values 0.150, 0.161, and 0.113 in the TSB, MB, and LB broth, respectively. All *L. monocytogenes* strains were capable of forming biofilms. Their strength depended on the incubation conditions. Only one strain (UB2-01-2 (1)) produced strong biofilm on TSB at 37 °C. Interestingly, the formation of biofilms did not occur at the temperature of 5 °C, although it is considered to be a survival strategy in adverse environmental conditions [40].

The adhesion of *L. monocytogenes* to contact surfaces is thought to be due to hydrophobic interactions between the surface material and the bacterial surface components. Flagella appear to play an important role in the initial adhesion [41]. Greater adhesion, which is a consequence of the difference in the structure of the flagella, was observed in serotype 1/2c compared to other serotypes [42], which is in agreement with the obtained results. Namely, isolate L5, which belongs to serotype 1/2c, moderately formed biofilms in the most different environmental conditions. Additionally, it is believed that the gene inlA (Internalin) causes the expression of the E-cadherin receptor, which participates in binding to the surface, and that the gene luxS (Luminescence) is a precursor to the process of biofilm formation and regulation [43]. Likewise, the virulence regulator PrfA (Positive Regulatory Factor A) promotes biofilm formation after the transition of the bacterium *L. monocytogenes* from the flagellar extracellular form to the surface-bound form [44].

At 37 °C, the flagellin expression was suppressed in most isolates of *L. monocytogenes* [45]. Nevertheless, *L. monocytogenes* is capable of passively binding to the surface [46], which is consistent with the results of this study and the findings of Kadam et al. [47], where the temperature of 37 °C was most favorable to the formation of biofilms as compared to lower temperatures. A similar effect was observed in other studies [48,49].

### 3.5. Biofilm Reduction

The exposure of 48-h-old *L. monocytogenes* biofilms on polystyrene to the most effective EOs (oregano, thyme, cinnamon, winter savory, and clove) at MBC for 48 h reduced *L. monocytogenes* biofilms in the range of 32.61% to 78.62% (Table 3). These results indicate the ability of EOs to penetrate into the biofilms and kill the protected bacterial cells.

However, the applied MBC values of EOs have a weaker effect on the cells within mature biofilms. The higher resistance of cells within mature biofilms was previously observed [50,51]. The resistance of bacteria in biofilms has been attributed to many factors. The negatively charged extracellular polysaccharide matrix, which coats the bacterial cells, represents a physical barrier that opposes the penetration of antimicrobial agents. In addition, protected cells in biofilms activate different genes than planktonic cells. Protected cells have the ability to inactivate antimicrobial peptides, as well as to activate efflux pumps that expel these agents from bacterial cells [51,52]. Mature biofilms are more tolerant to stressful conditions due to their stronger three-dimensional structure, which represents a physical barrier to antimicrobial agents [53].

### 3.6. SEM Observation

**Figure 3:**
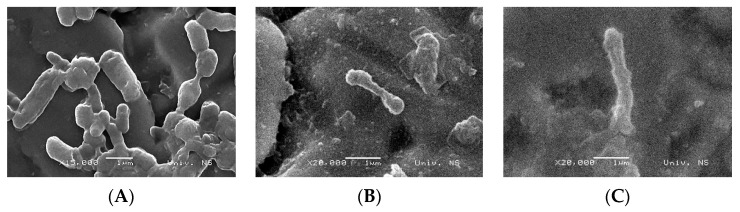
SEM images of *L. monocytogenes* cells: untreated (**A**) and treated with oregano (**B**) and thyme (**C**) essential oils.

SEM micrographs show the difference in the cell structure of untreated (control) bacteria and bacteria treated with oregano and thyme EOs (Figure 3). Untreated cells of *L. monocytogenes* have their typical structure. In contrast, bacterial cells treated with MIC values of oregano and thyme EOs underwent significant morphological changes. Both EOs caused the disruption of cell integrity and increased the permeability of cell membranes. Micrographs show cell membrane ruptures, the incomplete and deformed shape of bacterial cells, as well as cell lysis.

The phenolic compounds carvacrol and thymol, which are the main chemical components of oregano and thyme EOs, can integrate the bacterial cell membrane [31,54]. The accumulation of carvacrol or thymol within the membrane leads to the conformational change of the lipid bilayer, causing the disruption of normal membrane function, and an increase in membrane permeability. The hydroxyl group of these compounds and the presence of double bonds [55] allows the carvacrol and thymol to act as proton exchangers, leading to a decrease in the gradient across the cytoplasmic membrane, proton collapse, the inhibition of respiratory chain and electron transfer, and the oxidation and change of the pH gradient [56,57]. This leads to the loss of ATP and other cellular components, such as nucleic acids, ribosomes, lipids, proteins, and amino acids [58,59]. The death of bacterial cells can be a consequence of a large loss of cellular content or of the initiation of autolytic processes [56].

### 3.7. Microbiological Analysis of Minced Pork Meat

The inhibitory effects of oregano and thyme EOs on *L. monocytogenes* in minced pork meat samples stored at 4 °C for 4 days are shown in Table 4.

On days 0 and 1 of storage populations of *L. monocytogenes* in the control samples and the samples treated with oregano and thyme EOs were not different from each other (*p* > 0.05). The first significant (*p* < 0.05) decrease of *L. monocytogenes* was observed on day 2 in the samples treated with oregano EO at 0.36 µL/g, and with those treated with thyme EO at 0.36 µL/g and 0.72 µL/g. At the end of the experiment, the addition of oregano EO at 0.18 µL/g and 0.36 µL/g significantly (*p* < 0.05) reduced the number of *L. monocytogenes* by 0.25 log_10_ CFU/g and 0.51 log_10_ CFU/g, respectively. Similarly, the addition of thyme EO at 0.36 µL/g and 0.72 µL/g significantly (*p* < 0.05) reduced the number of *L. monocytogenes* by 0.22 log_10_ CFU/g and 0.29 log_10_ CFU/g, respectively. However, the population of *L. monocytogenes* increased in all treatments during storage. With respect to the use of oregano and thyme EOs, the present results are in agreement with those of Hulankova and Borilova [60].

Although in vitro studies show the excellent antibacterial effects of the oregano and thyme EOs, the amounts required in food models are usually higher than what is organoleptically acceptable [61]. Many researchers [33,60,62,63,64] believe that fats, proteins and carbohydrates absorb EOs and interfere with their mechanisms of action on bacterial cells. The higher availability of nutrients in the food matrix, compared to laboratory media, allows for the faster recovery of bacterial cells [65,66], while the lower water content of food reduces the antibacterial effect of EOs [62]. In addition, EOs that dissolve in the lipid phase of the food matrix are less available to act on bacteria found in the aqueous phase of the food [67].

## 4. Conclusions

In conclusion, the results of this study showed that 17 of 18 tested EOs have potential in vitro antilisterial activity. The ability to form biofilm is one of the survival strategies of *L. monocytogenes* in adverse environmental conditions. This study shows that *L. monocytogenes* strains can produce biofilm at 15 °C and 37 °C in TSB, MB, and LB broth on the polystyrene surface. The strength of the biofilm was found to be dependent on the availability of nutrients and on the temperature. Oregano, thyme, cinnamon, winter savory, and clove EOs showed the highest antilisterial activity, with MIC values > 0.09 µL/mL. The mentioned EOs reduced the biofilms of *L. monocytogenes* in the range of 32.61% to 78.62%. The growth inhibition of *L. monocytogenes* in minced pork meat was achieved with oregano and thyme EOs at MIC and 2MIC. These two EOs caused the disruption of cell integrity and the increased permeability of *L. monocytogenes* cell membranes. Micrographs, obtained by SEM, show cell membrane ruptures, and the incomplete and deformed shape of the *L. monocytogenes* cells. The application of these EOs in meat processing could be applied to efficiently reduce *L. monocytogenes* in meat products. However, further research on the acceptability of such meat products by consumers is needed.

## Figures and Tables

**Table 1:** The inhibition zones (expressed in mm) obtained testing the *L. monocytogenes* strains against 18 EOs (mean ± SD).

Essential Oils	L1		L5		L17		L18		UBL2–01-2(1)	
	5 µL	10 µL	5 µL	10 µL	5 µL	10 µL	5 µL	10 µL	5 µL	10 µL
Basil	- ^1^	8.68 ± 1.15 ^jk^	7.33 ± 0.58 ^gh^	10.33 ± 0.58 ^kl^	9.67 ± 0.58 ^de^	11.00 ± 3.61 ^ef^	9.67 ± 0.58 ^de^	13.00 ± 2.00 ^fg^	-	8.00 ± 0.00 ^i^
Black pepper	10.00 ± 0.00 ^de^	14.33 ± 1.15 ^gh^	7.00 ± 0.00 ^h^	16.33 ± 0.58 ^fg^	12.00 ± 1.00 ^cd^	14.33 ± 3.21 ^def^	12.00 ± 0.00 ^d^	15.00 ± 2.65 ^fg^	10.68 ± 0.58 ^ef^	15.00 ± 0.00 ^efg^
Cassumunar ginger	10.33 ± 1.15 ^de^	18.00 ± 0.00 ^e^	11.33 ± 1.15 ^e^	18.00 ± 2.00 ^f^	11.33 ± 0.58 ^d^	18.00 ± 2.00 ^d^	10.33 ± 0.58 ^de^	21.00 ± 1.00 ^d^	7.00 ± 0.00 ^g^	16.00 ± 1.73 ^ef^
Cinnamon	34.67 ± 1.15 ^b^	35.67 ± 1.15 ^c^	31.00 ± 1.00 ^c^	33.33 ± 1.15 ^d^	30.33 ± 0.58 ^b^	33.67 ± 1.53 ^c^	38.33 ± 1.53 ^a^	41.00 ± 1.73 ^c^	34.67 ± 0.58 ^b^	36.33 ± 2.31 ^c^
Clove	11.33 ± 1.15 ^d^	17.00 ± 1.00 ^ef^	15.67 ± 0.58 ^d^	16.67 ± 0.58 ^fg^	14.67 ± 1.53 ^c^	15.33 ± 2.52 ^def^	15.00 ± 0.00 ^c^	17.33 ± 1.15 ^def^	15.33 ± 1.15 ^d^	16.67 ± 1.15 ^e^
Curry plant	9.67 ± 1.53 ^de^	14.67 ± 0.58 ^gh^	10.33 ± 0.58 ^ef^	11.00 ± 1.00 ^jkl^	-	12.33 ± 1.53 ^ef^	9.00 ± 2.00 ^e^	15.33 ± 1.15 ^efg^	-	-
Fennel	-	-	-	-	-	-	-	-	-	-
Garden angelica	-	10.00 ± 0.00 ^ij^	-	9.00 ± 0.00 ^l^	-	11.33 ± 1.15 ^ef^	-	11.00 ± 0.00 ^g^	-	-
Hyssop	-	11.33 ± 1.15 ^i^	9.67 ± 0.58 ^ef^	21.33 ± 1.15 ^e^	10.00 ± 0.00 ^de^	13.33 ± 1.15 ^ef^	-	12.67 ± 0.58 ^fg^	8.67 ± 1.15 ^fg^	24.00 ± 2.00 ^d^
Lavender	9.00 ± 1.00 ^ef^	14.00 ± 1.73 ^gh^	10.00 ± 0.00 ^ef^	15.00 ± 0.00 ^gh^	9.00 ± 0.00 ^de^	13.33 ± 0.58 ^ef^	10.00 ± 0.00 ^de^	13.33 ± 1.15 ^fg^	8.00 ± 0.00 ^g^	13.33 ± 1.15 ^fg^
Lemon	7.67 ± 1.15 ^fg^	15.67 ± 0.58 ^fg^	11.00 ± 2.65 ^e^	16.33 ± 1.15 ^fg^	9.33 ± 1.53 ^de^	12.67 ± 3.06 ^ef^	10.00 ± 0.00 ^de^	14.33 ± 2.08 ^fg^	8.33 ± 1.53 ^fg^	15.67 ± 1.15 ^ef^
Myrtle	8.00 ± 0.00 ^fg^	11.67 ± 0.58 ^i^	9.00 ± 1.00 ^fg^	13.33 ± 0.58 ^hij^	11.67 ± 0.58 ^cd^	15.67 ± 1.15 ^de^	11.33 ± 0.58 ^de^	13.67 ± 0.58 ^fg^	11.67 ± 1.15 ^e^	13.67 ± 1.15 ^efg^
Oregano	40.00 ± 0.00 ^a^	43.67 ± 1.53 ^b^	40.33 ± 0.58 ^a^	42.67 ± 3.21 ^b^	37.33 ± 2.89 ^a^	40.67 ± 1.15 ^b^	37.67 ± 1.15 ^a^	46.00 ± 5.57 ^b^	34.00 ± 1.73 ^b^	35.33 ± 0.58 ^c^
Rosemary	7.00 ± 0.00 ^g^	8.00 ± 0.00 ^k^	7.67 ± 0.58 ^gh^	13.33 ± 1.15 ^hij^	-	-	10.67 ± 1.15 ^de^	20.00 ± 0.00 ^de^	8.00 ± 0.00 ^g^	10.33 ± 0.58 ^hi^
Sage	-	13.67 ± 1.53 ^h^	10.33 ± 1.15 ^ef^	14.67 ± 0.58 ^ghi^	11.33 ± 1.53 ^d^	12.33 ± 1.15 ^ef^	10.00 ± 0.00 ^de^	14.67 ± 0.58 ^fg^	8.33 ± 0.58 ^fg^	12.33 ± 0.58 ^gh^
Thyme	32.67 ± 1.53 ^c^	69.00 ± 1.00 ^a^	34.33 ± 0.58 ^b^	54.00 ± 2.65 ^a^	30.33 ± 4.93 ^b^	65.00 ± 5.57 ^a^	31.67 ± 4.62 ^b^	64.67 ± 8.08 ^a^	45.00 ± 3.61 ^a^	67.00 ± 2.65 ^a^
Winter savory	32.00 ± 0.00 ^c^	33.33 ± 1.53 ^d^	31.33 ± 0.58 ^c^	36.67 ± 2.08 ^c^	28.33 ± 0.58 ^b^	43.00 ± 2.65 ^b^	33.33 ± 0.58 ^b^	42.67 ± 2.31 ^bc^	30.33 ± 2.31 ^c^	39.67 ± 0.58 ^b^
Yarrow	10.33 ± 0.58 ^de^	16.67 ± 1.15 ^ef^	8.00 ± 0.00 ^gh^	12.33 ± 0.58 ^ijk^	7.00 ± 0.00 ^e^	10.67 ± 1.15 ^f^	-	11.00 ± 0.00 ^g^	8.00 ± 0.00 ^g^	15.00 ± 3.61 ^efg^

^1^ (-) A diameter of the inhibitory zone < 6 mm was considered as no antimicrobial activity. Values are the mean diameter of the inhibitory zone (mm) ± the SD of three replicates. Different letters in the column indicate statistically significant differences (*p* < 0.05).

**Table 2:** Biofilm formation on polystyrene plates with the three different growth media used at 5 °C, 15 °C, and 37 °C by *L. monocytogenes* strains tested in this study.

Strains	TSB			MB			LB		
	5 °C	15 °C	37 °C	5 °C	15 °C	37 °C	5 °C	15 °C	37 °C
L1	0.111 ± 0.010 º	0.221 ± 0.031 *	0.349 ± 0.046 **	0.121 ± 0.007 º	0.243 ± 0.051 *	0.290 ± 0.072 **	0.076 ± 0.007 º	0.124 ± 0.021 º	0.129 ± 0.022 *
L5	0.103 ± 0.006 º	0.584 ± 0.081 **	0.341 ± 0.047 **	0.138 ± 0.009 º	0.221 ± 0.038 *	0.324 ± 0,086 **	0.081 ± 0.009 º	0.442 ± 0.100 **	0.149 ± 0.010 *
L17	0.110 ± 0.008 º	0.144 ± 0.034 º	0.285 ± 0.037 **	0.116 ± 0.015 º	0.211 ± 0.075 *	0.279 ± 0.033 **	0.076 ± 0.010 º	0.139 ± 0.011 º	0.105 ± 0.012 º
L18	0.095 ± 0.007 º	0.124 ± 0.011 º	0.316 ± 0.070 **	0.095 ± 0.010 º	0.143 ± 0.025 º	0.232 ± 0.075 *	0.081 ± 0.008 º	0.122 ± 0.013 º	0.109 ± 0.015 º
UB2-01-2 (1)	0.111 ± 0.016 º	0.259 ± 0.030 *	0.519 ± 0.047 ***	0.122 ± 0.011 º	0.178 ± 0.024 *	0.242 ± 0.026 *	0.080 ± 0.011 º	0.382 ± 0.058 *	0.142 ± 0.018 *

Values are expressed as mean OD_550_ ± SD. Biofilm producer: ***: strong; **: moderate; *: weak; º: non biofilm producer.

**Table 3:** Reduction (%) of *L. monocytogenes* biofilms grown in polystyrene microtiter plate wells at 15 °C and 37 °C in TSB, MB, and LB broth with selected essential oils.

Strains	Growth Medium and Temperature	Essential Oils				
		Oregano	Thyme	Cinnamon	Winter Savory	Clove
L1	TSB 37 °C	51.72 ^a^	69.25 ^d^	57.16 ^ab^	64.92 ^cd^	61.44 ^bc^
L1	MB 37 °C	56.72 ^ab^	53.76 ^a^	59.20 ^bc^	62.64 ^c^	55.57 ^ab^
L5	TSB 37 °C	62.41 ^ab^	58.06 ^a^	59.70 ^a^	66.01 ^b^	64.76 ^b^
L5	TSB 15 °C	69.72 ^c^	66.32 ^b^	59.63 ^a^	66.05 ^b^	67.54 ^bc^
L5	MB 37 °C	46.58 ^b^	32.61 ^a^	42.90 ^b^	42.03 ^b^	41.02 ^b^
L5	LB 15 °C	78.62 ^b^	66.82 ^a^	77.85 ^b^	78.62 ^b^	76.75 ^b^
L17	TSB 37 °C	42.87 ^ab^	38.98 ^a^	44.01 ^ab^	51.64 ^b^	41.35 ^a^
L17	MB 37 °C	43.88 ^a^	46.86 ^ab^	51.28 ^b^	44.44 ^ab^	48.57 ^ab^
L18	TSB 37 °C	44.38 ^a^	41.24 ^a^	38.63 ^a^	40.77 ^a^	42.46 ^a^
UB2-01-2 (1)	TSB 37 °C	55.19 ^a^	54.32 ^a^	58.77 ^ab^	58.04 ^a^	63.31 ^b^

Values within the same row marked with different letters (a, b, c, d) in superscript indicate statistically significant differences (*p* < 0.05).

**Table 4:** Effects of oregano and thyme essential oils on *L. monocytogenes* (log_10_ CFU/g) in minced pork meat stored at 4 °C.

Treatments	Days				
	0	1	2	3	4
Control	4.18 ± 0.08 ^aD^	4.18 ± 0.06 ^aD^	4.56 ± 0.17 ^aC^	4.85 ± 0.07 ^aB^	5.28 ± 0.11 ^aA^
Oregano EO concentration 0.18 µL/g	4.20 ± 0.05 ^aC^	4.20 ± 0.14 ^aC^	4.54 ± 0.15 ^aB^	4.65 ± 0.17 ^bB^	5.03 ± 0.24 ^bA^
Oregano EO concentration 0.36 µL/g	4.23 ± 0.10 ^aC^	4.16 ± 0.10 ^aC^	4.30 ± 0.07 ^bC^	4.60 ± 0.13 ^bcB^	4.77 ± 0.15 ^cA^
Thyme EO concentration 0.36 µL/g	4.21 ± 0.04 ^aB^	4.10 ± 0.06 ^aC^	4.29 ± 0.03 ^bB^	4.42 ± 0.23 ^cdB^	5.06 ± 0.12 ^bA^
Thyme EO concentration 0.72 µL/g	4.19 ± 0.09 ^aC^	4.13 ± 0.08 ^aC^	4.14 ± 0.05 ^cC^	4.30 ± 0.10 ^dB^	4.99 ± 0.13 ^bA^

^a–d^ Statistically significant differences (*p* < 0.05) between the concentrations of essential oils. ^A–D^ Statistically significant differences (*p* < 0.05) between the days of storage.

## Data Availability

The data presented in this study are available on request from the corresponding author.

## Supplementary Materials

The following supporting information can be downloaded at: https://www.mdpi.com/article/10.3390/foods12101930/s1, Table S1: List of essential oils, their origin and expiration date.; Table S2: Chemical compositions (%) of the essential oils of basil (EO1), black pepper (EO2), cassumunar ginger (EO3), cinnamon (EO4), clove (EO5), and curry plant (EO6); Table S3: Chemical compositions (%) of the essential oils of fennel (EO7), garden angelica (EO8), hyssop (EO9), lavender (EO10), lemon (EO11), and myrtle (EO12); Table S4: Chemical compositions (%) of the essential oils of oregano (EO13), rosemary (EO14), sage (EO15), thyme (EO16), winter savory (EO17), and yarrow (EO18).
