# Supplementary material for: Effects of Selected Essential Oils on Listeria monocytogenes in Biofilms and in a Model Food System
Source: Foods. 2023 May 9;12(10):1930. doi: 10.3390/foods12101930 (PMC10217664; doi:10.3390/foods12101930)
Supplement: Supplementary file 1 [file foods-12-01930-s001.zip › foods-2311301-supplementary.pdf]

Supplementary Materials

Table S1. List of essential oils, their origin and expiration date.

No.	Essential Oils	Latin Name	Origin	Expiration date
1.	Basil	*Ocimum basilicum*	India	06.06.2019.
2.	Black pepper	*Piper nigrum*	India	22.12.2018.
3.	Cassumunar ginger	*Zingiber cassumunar*	India	31.10.2019.
4.	Cinnamon	*Cinnamomum zeylanicum nees*	Sri Lanka	31.10.2019.
5.	Clove	*Syzygium aromaticum L.*	India	30.11.2019.
6.	Curry plant	*Helichrysum italicum*	India	28.02.2020.
7.	Fennel	*Foeniculum vulgare*	India	31.03.2020.
8.	Garden angelica	*Angelica archangelica*	Serbia	08.2019.
9.	Hyssop	*Hyssopus officinalis*	Serbia	07.2021.
10.	Lavender	*Lavandula angustifolia*	India	29.09.2020.
11.	Lemon	*Citrus limonum*	India	28.02.2020.
12.	Myrtle	*Myrtus communis*	India	22.12.2018.
13.	Oregano	*Origanum vulgare*	India	28.02.2020.
14.	Rosemary	*Rosmarinus officinalis*	Spain	30.11.2019.
15.	Sage	*Salvia officinalis*	India	29.09.2020.
16.	Thyme	*Thymus vulgaris*	India	07.2019.
17.	Winter savory	*Satureja montana*	Serbia	07.2020.
18.	Yarrow	*Achillea millefolium*	Serbia	10.2020.

Table S2. Chemical compositions (%) of the essential oils of basil (EO1), black pepper (EO2), cassumunar ginger (EO3), cinnamon (EO4), clove (EO5), and curry plant (EO6).

No.	Compounds	RI	EO1	EO2	EO3	EO4	EO5	EO6
1.	α-Thujene	926	-	1.82	-	-	-	0.04
2.	α-Pinene	932	-	10.75	1.13	0.89	-	3.77
3.	Camphene	942	-	0.18	-	0.40	-	0.06
4.	Benzaldehyde	950	-	-	-	4.34	-	-
5.	Sabinene	960	-	13.55	38.17	-	-	-
6.	β-Pinene	963	-	19.31	8.23	0.40	-	0.09
7.	Sulcatone	969	0.04	-	-	-	-	-
8.	β-Myrcene	972	-	0.48	-	-	-	0.19
9.	α-Phellandrene	985	-	0.36	0.11	-	-	0.30
10.	3-Carene	990	-	7.49	-	-	-	-
11.	α-Terpinene	995	-	8.08	1.04	-	-	-
12.	*p*-Cymene	1003	-	2.52	2.75	1.15	-	0.43
13.	Limonene	1007	-	13.93	0.28	0.61	-	1.48
14.	1,8-Cineole	1009	0.24	0.61	0.80	0.23	-	-
15.	β-Ocimene	1023	0.08	-	-	-	-	-
16.	γ-Terpinene	1034	-	0.85	10.06	0.07	-	0.27
17.	α-Terpinolene	1063	-	0.59	0.47	-	-	0.45
18.	Linalool	1075	24.77	1.01	-	1.83	-	1.21
19.	Limona ketone	1108	-	-	-	-	-	0.23
20.	Dihydrolinalool	1109	0.13	-	-	-	-	-
21.	Camphor	1123	-	0.12	0.09	-	-	-
22.	Menthone	1132	-	0.29	-	-	-	-
23.	Isoborneol	1136	-	-	-	0.25	-	-

24.	Acetic acid, phenylmethyl ester	1143	-	-	-	0.96	-	-
25.	dl-Menthol	1152	0.23	0.32	-	-	-	-
26.	Terpinen-4-ol	1158	-	1.03	35.90	0.10	-	-
27.	α-Terpineol	1173	1.30	0.08	0.39	-	-	-
28.	Estragole	1183	69.52	-	-	-	-	-
29.	Citronellol	1213	-	0.23	-	-	-	-
30.	Nerol	1214	-	-	-	-	-	3.66
31.	α-Citral	1228	0.22	-	-	-	-	-
32.	Carvone	1231	-	0.30	-	-	-	-
33.	Chavicol	1240	-	-	-	-	0.06	-
34.	Geraniol	1242	-	0.19	-	-	-	0.08
35.	β-Citral	1261	0.30	-	-	-	-	-
36.	Cinnamaldehyde	1271	-	-	-	74.93	-	-
37.	Safrole	1283	-	-	-	0.06	-	-
38.	α-Cubebene	1348	-	0.35	-	-	-	0.27
39.	Eugenol	1358	-	0.96	-	3.10	85.14	0.09
40.	Neryl acetate	1365	-	-	-	-	-	18.15
41.	Copaene	1378	-	3.74	-	-	0.11	0.65
42.	Geranyl acetate	1385	-	-	-	-	-	0.11
43.	Germacrene D	1393	-	0.23	-	-	-	-
44.	Vanillin	1400	-	-	-	0.24	-	-
45.	Longifolene	1409	-	0.98	-	-	-	0.34
46.	α-Gurjunene	1414	-	-	-	0.13	-	-
47.	Caryophyllene	1425	0.32	7.15	-	0.15	10.20	21.48
48.	β-Cubebene	1435	-	-	-	-	-	0.17
49.	α-Bergamotene	1441	0.52	-	-	-	-	1.33
50.	Cinnamyl acetate	1450	-	-	-	0.05	-	-
51.	α-Himachalene	1457	-	-	-	-	-	5.34
52.	α-Humulene	1460	0.17	0.23	0.07	-	2.64	3.08
53.	β-Farnesene	1463	0.18	-	-	-	-	-
54.	γ-Himachalene	1487	-	-	-	-	-	3.34
55.	α-Cedrene	1490	-	-	-	-	-	0.83
56.	Germacrene	1490	0.26	-	-	-	-	-
57.	β-Himachalene	1512	-	-	-	-	-	13.34
58.	β-Bisabolene	1517	0.06	-	-	-	-	0.19
59.	Calamenene	1532	-	-	0.16	-	-	-
60.	δ-Cadinene	1533	-	0.59	-	-	0.23	0.30
61.	Acetyleugenol	1536	-	-	-	0.05	0.21	-
62.	α-Bisabolene	1552	1.43	-	-	-	-	0.70
63.	Elemol	1559	-	-	-	-	-	0.16
64.	Nerolidol	1573	-	-	-	-	-	0.30
65.	*p*-Methoxycinnamaldehyde	1576	0.22	-	-	-	-	-
66.	Caryophyllene oxide	1593	-	0.11	-	-	0.82	0.74
67.	Longiborneol	1606	-	-	-	-	-	0.16
68.	Humulene epoxide	1619	-	-	-	-	0.10	-
69.	γ-Eudesmol	1640	-	-	-	-	-	0.43
70.	Thujopsene	1647	-	-	-	-	-	0.19
71.	α-Cedrene	1656	-	-	-	-	-	0.56
72.	β-Eudesmol	1658	-	-	-	-	-	0.34

73.	Ascabin	1764	-	0.07	-	9.01	-	-
74.	α-Atlantone	1775	-	-	-	-	-	4.76
	**Total**		**100.00**	**98.52**	**99.64**	**98.96**	**99.50**	**89.62**

Table S3. Chemical compositions (%) of the essential oils of fennel (EO7), garden angelica (EO8), hyssop (EO9), lavender (EO10), lemon (EO11), and myrtle (EO12).

No.	Compounds	RI	EO7	EO8	EO9	EO10	EO11	EO12
1.	α-Thujene	926	-	1.64	0.37	-	-	0.31
2.	α-Pinene	932	0.24	13.27	1.01	1.36	1.41	35.47
3.	Camphene	942	-	8.72	0.17	0.07	0.17	0.44
4.	Sabinene	960	-	1.68	1.97	-	0.31	-
5.	β-Pinene	963	-	0.80	13.16	0.63	1.11	1.02
6.	Dihydrocamphene	963	0.04	-	-	-	-	-
7.	3-Octanone	968	-	-	-	0.65	-	-
8.	β-Myrcene	972	-	3.54	1.51	0.30	1.54	0.24
9.	3-Octanol	975	-	-	-	0.15	-	-
10.	Octanal	982	-	-	-	-	0.11	-
11.	α-Phelandren	985	-	4.53	-	-	-	-
12.	3-Carene	990	-	4.59	-	0.43	4.11	8.80
13.	α-Terpinene	995	-	-	0.38	-	-	0.22
14.	*p*-Cymene	1003	0.18	2.37	0.27	1.65	0.28	2.41
15.	Limonene	1007	2.13	-	-	2.72	79.72	0.56
16.	β-Phelandren	1007	-	41.57	5.58	-	-	-
17.	1,8-Cineole	1009	-	-	0.47	1.13	-	19.58
18.	α-Ocimene	1013	-	0.37	0.13	-	-	-
19.	β-Ocimene	1023	-	0.84	0.47	0.04	-	-
20.	γ-Terpinene	1034	0.07	0.20	0.64	-	0.12	1.13
21.	Linalool oxide	1048	-	-	-	0.59	-	-
22.	α-Terpinolene	1063	0.05	0.28	0.19	-	0.45	0.79
23.	Phenone	1064	1.24	-	-	-	-	-
24.	Linalool	1075	-	-	2.97	23.88	0.82	6.28
25.	β-Thujone	1082	-	-	0.11	-	-	-
26.	cis-Limonene oxide	1111	-	-	-	-	0.32	-
27.	trans-Limonene oxide	1115	-	-	-	-	0.11	-
28.	Camphor	1123	0.06	-	0.10	2.82	-	0.32
29.	Menthone	1132	-	-	-	-	-	0.15
30.	Isoborneol	1136	-	-	-	1.73	0.09	-
31.	trans-Pinocamphone	1141	-	-	7.44	-	-	-
32.	endo-Borneol	1145	-	-	-	0.12	-	-
33.	dl-Menthol	1152	-	-	-	-	-	0.27
34.	Isononyl acetate	1155	-	-	-	10.05	-	-
35.	Terpinen-4-ol	1158	-	0.19	-	-	-	0.85
36.	cis-Pinocamphone	1159	-	-	27.42	-	-	-
37.	Cryptone	1168	-	1.00	-	-	-	-
38.	α-Terpineol	1173	-	-	0.18	1.71	1.47	5.02
39.	γ-Terpineol	1180	-	-	-	0.27	-	0.88
40.	Estragole	1183	2.97	-	4.13	-	-	-
41.	Decanal	1188	-	-	-	-	0.20	-
42.	Carveol	1203	-	-	-	-	0.10	-
43.	Citronellol	1213	-	-	-	-	0.07	-

44.	Phenyl acetate	1220	0.12	-	-	-	-	-
45.	Cumaldehyde	1227	-	0.22	-	-	-	-
46.	α-Citral	1228	-	-	-	-	1.49	-
47.	Carvone	1231	-	-	-	-	0.20	0.42
48.	Geraniol	1242	-	-	0.21	-	0.10	0.40
49.	Anisaldehyde	1243	0.72	-	-	-	-	-
50.	Linalyl acetate	1246	-	-	-	25.33	-	0.87
51.	β-Citral	1261	-	-	-	-	1.75	-
52.	Borneol acetate	1279	-	0.47	-	1.84	-	-
53.	Cuminol	1282	-	0.14	-	-	-	-
54.	Anethole	1288	88.42	-	-	-	-	-
55.	Limonene dioxide	1299	-	-	-	-	-	1.52
56.	α-Terpinyl acetate	1349	-	-	-	12.58	-	-
57.	Citronellol acetate	1351	-	-	-	-	0.40	-
58.	Eugenol	1358	-	-	0.21	-	-	0.61
59.	Neryl acetate	1365	-	-	-	0.29	-	-
60.	Cyclosativene	1368	-	0.19	-	-	-	-
61.	Copaene	1378	-	2.08	0.19	0.11	-	0.76
62.	Geranyl acetate	1385	-	-	-	0.05	0.10	0.60
63.	Anisic ketone	1385	0.10	-	-	-	-	-
64.	β-Bourbonene	1387	-	-	2.19	-	-	-
65.	β-Elemene	1395	-	0.31	1.40	-	-	-
66.	Methyl eugenol	1408	-	-	0.26	-	-	-
67.	Longifolene	1409	-	-	-	0.06	-	0.88
68.	α-Gurjunene	1414	-	-	0.27	-	-	-
69.	Caryophyllene	1425	-	-	3.35	5.21	1.98	3.79
70.	β-Cubebene	1435	-	0.36	0.24	-	-	-
71.	α-Bergamotene	1441	-	-	0.32	-	-	-
72.	Coumarin	1441	-	-	-	1.43	-	-
73.	α-Guaiene	1444	-	-	0.87	-	-	-
74.	α-Humulene	1460	-	0.41	1.09	0.57	-	0.49
75.	Aromandendrene	1468	-	-	1.33	-	-	-
76.	Germacrene D	1490	-	-	4.50	-	-	-
77.	Curcumene	1490	-	0.82	-	-	-	-
78.	Zingiberene	1504	-	0.59	-	-	-	-
79.	Bicyclogermacrene	1506	-	-	2.19	-	-	-
80.	α-Muurolene	1509	-	0.65	-	-	-	-
81.	α-Bulnesene	1515	-	-	2.27	-	-	-
82.	β-Bisabolene	1517	-	0.48	-	-	-	-
83.	γ-Cadinene	1523	-	-	1.10	-	-	-
84.	δ-Cadinene	1533	-	0.40	0.24	0.07	-	-
85.	α-Copaen-11-ol	1551	-	0.28	-	-	-	-
86.	Elemol	1559	-	-	1.22	-	-	-
87.	Spathulenol	1587	-	0.59	0.37	-	-	-
88.	Caryophyllene oxide	1593	-	-	0.87	0.21	0.33	-
89.	Ledol	1613	-	-	0.10	-	-	-
90.	3-Decanolide	1636	-	0.89	-	-	-	-
91.	γ-Eudesmol	1640	-	-	0.20	-	-	-
92.	T-Muurolol	1648	-	-	1.34	-	-	-
93.	β-Eudesmol	1658	-	-	0.23	-	-	-
94.	α-Eudesmol	1661	-	-	0.21	-	-	-

95.	Pentadecanolide	1822	-	0.31	-	-	-	-
	**Total**		**96.35**	**94.79**	**95.44**	**98.05**	**98.85**	**95.11**

Table S4. Chemical compositions (%) of the essential oils of oregano (EO13), rosemary (EO14), sage (EO15), thyme (EO16), winter savory (EO17), and yarrow (EO18).

No.	Compounds	RI	EO13	EO14	EO15	EO16	EO17	EO18
1.	Santolina triene	912	-	-	-	-	-	0.21
2.	α-Thujene	926	-	0.12	-	0.06	1.79	0.55
3.	α-Pinene	932	0.29	28.23	-	0.40	1.06	2.28
4.	Camphene	942	0.05	3.44	-	0.07	0.43	0.20
5.	Sabinene	960	-	-	-	-	-	22.70
6.	1-Octen-3-ol	962	-	-	-	-	1.37	-
7.	β-Pinene	963	0.19	3.16	-	1.28	-	11.47
8.	β-Myrcene	972	0.09	0.16	0.96	0.64	1.33	0.60
9.	α-Phelandren	985	-	0.06	0.35	0.18	0.29	0.10
10.	3-Carene	990	-	0.22	-	-	0.08	-
11.	α-Terpinene	995	-	-	-	-	2.26	0.63
12.	*p*-Cymene	1003	1.61	2.04	0.16	40.91	15.73	0.37
13.	Limonene	1007	0.14	3.43	0.94	0.39	0.59	-
14.	β-Phelandren	1007	-	-	-	-	-	1.00
15.	1,8-Cineole	1009	0.23	11.54	-	0.06	0.33	3.06
16.	α-Ocimene	1013	-	-	0.29	-	-	0.20
17.	o-Cymene	1015	-	-	-	0.07	-	-
18.	β-Ocimene	1023	-	-	-	-	-	0.51
19.	γ-Terpinene	1034	0.13	0.34	-	10.37	11.43	-
20.	Artemisia ketone	1036	-	-	-	-	-	7.37
21.	Artemisia alcohol	1057	-	-	-	-	-	0.17
22.	α-Terpinolene	1063	-	0.36	0.49	-	0.16	0.27
23.	Linalool	1075	0.18	1.03	23.95	2.18	1.06	0.37
24.	iso-Phenol	1085	-	0.46	-	-	-	-
25.	Dihydrolinalool	1109	-	-	0.32	-	-	-
26.	Camphor	1123	0.10	1.23	-	-	-	0.32
27.	Borneol	1139	-	24.87	-	-	-	-
28.	endo-Borneol	1145	-	0.31	0.10	-	1.26	-
29.	Terpinen-4-ol	1158	-	0.65	-	0.76	0.81	2.11
30.	α-Terpineol	1173	0.24	11.86	4.77	-	0.18	0.33
31.	γ-Terpineol	1180	-	1.57	0.66	-	-	-
32.	Nerol	1214	-	-	0.08	-	-	-
33.	Geraniol	1242	-	0.45	-	-	-	-
34.	Linalyl acetate	1246	-	0.21	56.41	-	-	-
35.	Borneol acetate	1279	-	1.30	-	-	-	-
36.	Lavandulyl acetate	1283	-	-	-	-	-	0.42
37.	Thymol	1286	7.74	-	-	40.36	0.28	-
38.	Carvacrol	1299	81.00	-	-	1.44	50.45	0.44
39.	α-Terpinyl acetate	1349	-	1.23	-	-	-	-
40.	Neryl acetate	1365	-	-	1.51	-	0.10	0.32
41.	Carvacryl acetate	1373	-	-	-	-	0.44	-
42.	Copaene	1378	0.13	-	-	-	0.13	0.17
43.	Geranyl acetate	1385	-	-	2.29	-	-	-
44.	β-Bourbonene	1387	-	-	-	-	0.11	0.55

45.	β-Curcumene	1407	-	-	-	-	-	0.11
46.	Longifolene	1409	-	0.25	0.10	-	-	-
47.	Caryophyllene	1425	6.69	0.63	2.13	-	2.68	11.69
48.	α-Humulene	1460	0.66	0.07	0.35	-	0.10	1.73
49.	β-Farnesene	1463	-	-	-	-	-	0.14
50.	γ-Muurolene	1485	-	-	-	-	0.19	-
51.	Germacrene D	1490	-	-	-	-	1.39	14.10
52.	β-Selinene	1495	-	-	-	-	0.14	0.20
53.	Zingiberene	1504	-	-	-	-	-	0.92
54.	Bicyclogermacrene	1506	-	-	-	-	0.57	0.54
55.	Farnesene	1517	-	-	-	-	-	0.25
56.	β-Bisabolene	1517	-	-	-	-	1.44	-
57.	γ-Cadinene	1523	-	-	-	-	0.14	-
58.	Calamenene	1532	-	-	0.72	-	-	-
59.	δ-Cadinene	1533	0.09	-	-	-	0.30	0.55
60.	Cadina-1,4-diene	1541	-	-	0.07	-	-	-
61.	Nerolidol	1573	-	-	-	-	-	0.32
62.	Caryophyllene oxide	1593	0.31	-	0.41	-	0.33	1.81
63.	β-Eudesmol	1658	-	-	-	-	-	0.13
64.	Chamazulene	1734	-	-	-	-	-	6.70
	**Total**		**99.87**	**99.19**	**97.06**	**99.17**	**98.94**	**95.91**
